# The parallels between particle induced lung overload and particle induced periprosthetic osteolysis (PPOL)

**DOI:** 10.12688/openreseurope.13264.1

**Published:** 2021-03-24

**Authors:** Paul J.A. Borm

**Affiliations:** 1 Nanoconsult, Meerssen, 6231 CV, The Netherlands; 2 University of Dusseldorf, Dusseldorf, 50224, Germany

**Keywords:** osteolysis, particle overload, fibrosis, PSLT, hip implants, microplastics

## Abstract

**Background:** When particles deposit for instance in the lung after inhalation or in the hip joint after local release from a hip implant material they can initiate a defense response. Even though these particles originate from inert materials such as polyethylene (PE) or titanium, they may cause harm when reaching high local doses and overwhelming local defense mechanisms.

**Main body:** This paper describes the parallels between adverse outcome pathways (AOP) and particle properties in lung overload and periprosthetic osteolysis (PPOL). It is noted that in both outcomes in different organs , the macrophage and cytokine orchestrated persistent inflammation is the common driver of events, in the bone leading to loss of bone density and structure, and in the lung leading to fibrosis and cancer. Most evidence on lung overload and its AOP is derived from chronic inhalation studies in rats, and the relevance to man is questioned. In PPOL, the paradigms and metrics are based on human clinical data, with additional insights generated from *in vitro* and animal studies. In both organ pathologies the total volume of particle deposition has been used to set threshold values for the onset of pathological alterations. The estimated clinical threshold for PPOL of 130 mg/ml is much higher than the amount to cause lung overload in the rat (10 mg/ml).

**Conclusions**: The paradigms developed in two very different areas of particle response in the human body have major similarities in their AOP. Connecting the clinical evidence in PPOL to lung overload challenges current paradigms and the human relevance of rat inhalation studies.

## Plain language summary

When walking the stairs, dancing or doing groceries we use our hips. When growing older many people need a new hip, since dysfunction of a hip causes major discomfort, pain and reduces quality of life. A new hip prosthesis can be inserted surgically to restore normal function and this procedure is nowadays very common as the average age of population increases. The lifetime of a new hip is determined by the wear rate of the materials in the moving part. These little particles may lead to a dysfunctional hip joint. This phenomenon is described by doctors as hip particle disease. The lung is our most important organ for supply of oxygen to the blood and very vulnerable to high amounts of particle inhalation such as in dusty jobs such as former coal mining. Interestingly, the lung reacts to particle presence in the same standard way as the bone. Upon a certain dose of particles, both in lung and bone an inflammatory response is noted that leads to scar tissue in the lung and bone damage in the joint. The nature of the materials and particles is less important but only the dose counts, until a certain threshold is exceeded. This paper connects these two worlds of science and gives some hints for both doctors in lung disease and orthopedics how to use each other’s know-how and learning curves. As opposed to hip implant, lung transplantation is not a routine option to replace a diseased lung; on the other hand, hip transplantation is among the top five surgical procedures worldwide and the paradigms developed after abundant and long-term use of this clinical procedure may be useful to extrapolate to the lung.

## Introduction

Inflammatory diseases can affect many organs and can come in different manifestations including rheumatoid arthritis (RA), Crohn’s disease and asthma. Chronic inflammation plays a key role in the etiology and progression of many medical conditions ( Furman *et al.*, 2019). Inflammation and the immune cells involved influence the activities of local tissue cells, often leading to local damage and tissue repair and remodeling ( Weitzman *et al.*, 1985). In two specific target area’s inflammation is driven by a high local load of inert particles, that is the lung (by inhalation of particles) and the bone in case an internal hip implant releases large amounts of debris particles.

More than 30 years ago Morrow (1988) observed that particle retention increased in rats when they inhaled high concentrations of insoluble particles during a longer time period. Under such conditions, rats also developed lung fibrosis and lung cancer after lifetime exposure. This observation led to the understanding that any poorly soluble low toxicity particles (PSLT) can cause lung cancer in the rat, provided exposure is sufficiently high. This has been confirmed for materials as diverse as carbon black, TiO _2_, toner, diesel, talc and iron oxides, as well as polymer particles. The process is now commonly described as “lung particle overload” and in the rat this phenomenon can be recognized by a sequence of events, including accumulation of particle laden macrophages, persistent neutrophilic inflammation, epithelial hyperplasia, metaplasia, lung fibrosis and formation of lung tumors ( Borm *et al.*, 2015; ECETOC, 2013). Whereas originally volumetric load of the lung and macrophages was used as a metric to describe dose-rate and clearance retardation, it is now recognized that particle surface dose rate may be a better descriptor of inflammation and subsequent genotoxic responses ( Driscoll *et al.*, 1997; Driscoll & Borm, 2020)

Metal alloys and polymer materials have been used for more than 50 years to replace hip segments such as the femoral stem and acetabular shell. The articular surface on the femoral side is usually substituted by parts made of metal alloys or ceramic. Various forms of polyethylene (PE) are used to for the articular surface on the acetabular side ( Kandahari *et al.*, 2016). In the orthopedics community particles generated from artificial hip implants during movement which are then released into the synovial fluid have been described for decades ( Dumbleton *et al.*, 2002; Gallo *et al.*, 2014). The release of particles from different implant materials is a continuous process and known to lead after time to an inflammatory response in the synovial fluid and peri-prosthetic bone ( Goodman & Gallo, 2019; Mihalko *et al.*, 2020). While low wearing hip implants were predicted to stay osteolysis-free, hip implants with a higher wear rate would inevitably lead to osteolysis ( Mbaviele *et al.*, 2017). Recent clinical reviews ( Elke & Rieker, 2018) have estimated and quantified a particle volume of approximately 670 mm ^3^ as the osteolysis threshold after total hip arthroplasty (THA). Beyond this volumetric threshold, osteolysis is known to start and affect function and structure of the peri-prosthetic bone.

This review sets out to compare the AOP underlying rat lung fibrosis and cancer in lung particle overload in rats and humans, and that for wear induced osteolysis (PPOL) from artificial hip implants. Doing so we focus on inert particle wear for the purpose of making the comparison based on inherent particle characteristics such as volume, size, surface and numbers. This cross-disciplinary effort is then continued to compare the human relevance of the paradigms developed in both endpoints.

## PSLT and Lung overload- current paradigms

Lifetime rat inhalation studies with different particles including titanium dioxide, carbon black, but also talc and toner ( Heinrich *et al.*, 1995 and Heinrich *et al.*, 1986) showed that these so-called inert particles of low inherent toxicity (PSLT) can cause both lung fibrosis and lung tumors. This lung response is independent of particle type and is characterized by an accumulation of particle laden macrophages, persistent neutrophilic inflammation, epithelial hyperplasia, metaplasia and interstitial fibrosis ( Borm *et al.*, 2015; Driscoll & Borm, 2020). Many inhaled particles induce *in vitro* and *in vivo* reactive oxygen species (ROS) production (review: Ma, 2020) as well as downstream inflammatory effects (reviews: Ma, 2020; Riediker *et al.*, 2019). Apart from producing ROS, inflammatory phagocytes (M1) and structural cells produce growth factors, cytokines and chemokines upon contact with particles ( Bissonnette *et al.*, 2020) to maintain lung homeostasis. After a phase of acute inflammation resolution and tissue repair is observed which involves type 2 cytokines, M2 macrophages, Th2 lymphocytes and is orchestrated by so-called specialized pro-resolving mediators (SPMs). Resolution of inflammation is often disturbed and incomplete due to the presence of particulate matter in the tissue which can lead to persistent type 2 inflammation. In long term this remodeling can lead to interstitial fibrosis, granuloma and tumor formation ( Ma, 2020). Recent studies also reveal the involvement of Th17-, T-reg and other response in the subsequent pathogenesis caused by inhaled particulates and is considered a long term immune response playing a role in the long-term outcome of particle induced inflammation ( Ma, 2020).

The fact that any sufficiently high dose of inert particles is able to cause lung fibrosis and cancer in the rat was denominated as lung particle overload ( Morrow, 1988). Both Morrow (1988) and many sequel studies demonstrated that a specific threshold related to the volume of particles phagocytized by alveolar macrophages needs to be exceeded before the acute and chronic effects are initiated. This threshold was suggested to be directly connected to the volume of the macrophage population leading to impaired macrophage function after loading a certain fraction of this clearance cell system ( Oberdörster, 1996). Morrow’s original theory was simple and stated that beyond a certain cellular uptake of particles by the alveolar macrophage (around 1/6th of it volume) clearance will be impaired and retained lung burden will no longer be able to reach a steady-state but increase with exposure. He also showed a connection between fractional elimination rate (/day) and the retained particle lung burden both in volume (µl) and in surface dose (cm ^2^). Decrease of elimination rate had already started at 0.1 µl ( Morrow, 1988) or 200 cm ^2^ ( Tran *et al.*, 2000) and complete inhibition of clearance was present at 10 µl (= 10 mm ^3^) per gram of lung. This lung overload phenomenon is considered to be a threshold phenomenon, which has implication for both hazard and risk assessment of inhaled particles. Despite the considerable research which has been undertaken on the lung particle overload phenomenon debate continues until today regarding the underlying mechanism(s), species similarities and differences ( Driscoll & Borm, 2020).

## Wear rate and osteolysis- current paradigms

Periprosthetic osteolysis (PPOL) is commonly regarded as the adverse outcome as a response to an artificial hip implant that determines its lifetime. PPOL is considered to be caused by the cellular response of the human bone to wear debris. Any type and size of particle (polymer, ceramic, or metal) is suggested to be a hazard for osteolysis, hence the alternative term *particle disease* was introduced early to describe this phenomenon ( Dumbleton *et al.*, 2002; Goodman *et al.*, 2020). Wear particles can be induced by different physical and chemical processes including wear, but also due to abrasive and third body actions, fatigue of the material, and corrosion or brittle materials. Although pressure differences have been shown to play a role in PPOL ( van der Vis *et al.*, 1998), it is more prevalent in the presence of wear. Numerous responses induced by wear particles on osteoclasts have led to the understanding that this cell is the most important effector cell in PPOL. In addition it is generally accepted that particles smaller than 1 micrometer are most likely to cause the osteolytic response, but this evidence is mainly based on *in vitro* studies (review: Gibon *et al.*, 2017; Mbaviele *et al.*, 2017; Nine *et al.*, 2014).

Before market introduction and clinical introduction, wear rate of total hip joint prosthesis is to be measured according to ISO14242-1, 2 and 3 and is part of the submission file to notified bodies. The osteolysis free lifetime is actually calculated from the volumetric wear rate as:

*osteolysis free life = osteolysis threshold (mm ^3^) /volumetric wear rate (mm ^3^/year)*

It is generally accepted that a threshold for periprosthetic osteolysis exists, and its value is based on a series of clinical observations and derived from long-term wear, which is clinically assessed as penetration of the head into the cup liner using 2D anteroposterior radiographs. There are two different ways to define an osteolysis threshold, either using a linear wear rate (in mm/year) or a total accumulated wear volume (mm ^3^ or ml). Dumbleton *et al.* (2002) stated that osteolysis occurs rarely when linear wear rates are between 0.05 and 0.1 mm/year. A more recent clinical review ( Elke & Rieker, 2018) assessed the total wear volume to assess the osteolysis free life of total hip arthroplasty. They concluded that a cumulative wear volume of 670 mm ^3^ (range 590–800) of polyethylene particles will induce osteolysis. The narrow range between 590 and 800 mm ^3^ points to a well-controlled and characteristic response of the human body to these UHMWPE or other types of wear particles ( Merola & Affatato, 2019).

Particles and their constituents generated by wear from hip or knee can also reach local lymph nodes as well as the spleen, liver and kidneys ( Gallo *et al.*, 2014; Nine *et al.*, 2014; Revell, 2008). Wherever particles accumulate, a granulomatous reaction characterized by multinucleated giant cells and macrophages containing particles, lymphocytes and other inflammatory cells may occur, depending on the number of particles and their size ( Goodman & Gallo, 2019). In addition to this a type IV cell-mediated immune reaction may occur, when TH1 cell response and active antigen presentation are involved ( Nich *et al.*, 2016; Revell, 2008). Such reactions are mostly seen in failed arthroplasties with a very high load of wear debris. They occur mostly in close proximity to the implant, but may also be found with decreasing incidence and severity in local lymph nodes, and occasionally in remote organs, such as the liver.

## Particle induced pathology in lung and bone- a common mechanism of action?

Both lung and bone and lung develop a response to particle wear or deposition which in bone leads to degradation of the bone (periprosthetic osteolysis, PPOL) and in the lung to increased proliferation and fibrosis. Similar mediators and effectors cells are involved in a process that seems to be initiated by a certain volumetric particle load and more or less independent from the used particle material. Studies on osteolysis have been driven and fed by clinical performance of implants and the quest for better materials with less wear particles, increasing the functional lifetime of implants ( Goodman *et al.*, 2020; Mihalko *et al.*, 2020). Particles are generated from hard, durable and biocompatible materials such as HMWPE, metals, ceramics and PEEK that have been around for a few decades, and the transition to new materials has been slow due to strong demands on functional testing and clinical evaluation. New (nano) materials and structures, to reduce wear and at the same time provide anti-microbial protection are available but barely being used in current products ( Mihalko *et al.*, 2020). In addition, new production methods such 3D printing can fabricate personalized organs/scaffolds that are capable of biomimicking the delicate structures and surfaces of the bone components ( Okolie *et al.*, 2020). These applications apply new (bio)materials such as collagen, nano Ca-Phosphate, cellulose, PLGA, and alginates that are needed for hard/soft tissue engineering. It is anticipated that with these structures and materials, interfaces are made which are less prone to release of particles, or debris that shows less immunogenic or inflammatory response ( Albrektsson & Wenneberg, 2019).

In lung toxicology, the nuisance particle issue more or less disappeared from the fore due to successful fight against occupational particle induced diseases such as coal worker’s pneumoconiosis or asbestosis. However, in recent years the toxicology of so-called particles with no inherent toxicity (PSLT) has become extremely important with regard to the classification of materials within the framework of CLP. The recent substance classification of titanium dioxide as a class II carcinogen ( ECHA, 2017) has illustrated the limitations hazard based CLP legislation ( Riebeling *et al.*, 2020). It is expected that other low-toxicity particulate materials will follow the same classification and ban from value chains in classic industry, concerning pigments, cosmetics, excipients and many other products.

**Figure 1.**
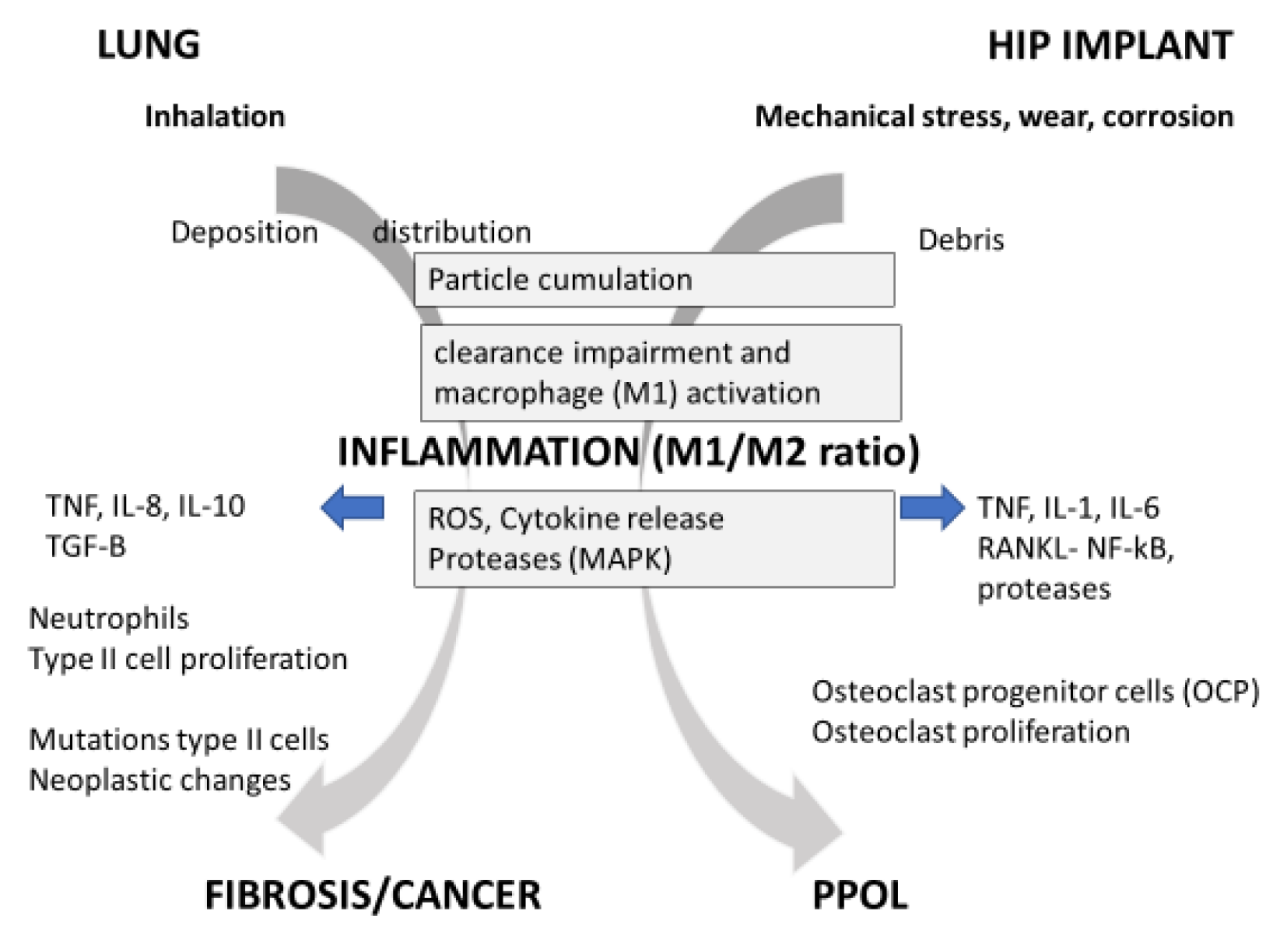
Schematic presentation of adverse outcome pathway of particle exposure in lung and bone, showing the common pathways, mediators and cells caused by particle deposition and impaired clearance leading to inflammation. However, depending on environment and target different effector cells and endpoints are being induced. *Abbreviations: TNF-α, tumor necrosis factor-alpha; TGF-Beta: transforming growth factor-beta; IL-1, IL-6, IL-8 IL-18: Interleukins 1,6, 8 and 18 ; MMP, metallo-proteinases; MAPs, mitogen activated protein kinases; RANKL, receptor activator of nuclear factor kappa-B ligand ;NF-κB, Nuclear Factor- Kappa B; AP-1, Activator protein-1; PPOL is pre-prosthetic osteolysis. M1 and M2 are different types of macrophages.*

Both in lung fibrosis and periprosthetic osteolysis (PPOL) it is recognized that accumulation of particles either from inhalation or released wear is crucial. At a certain burden of particles, the normal biological response will not be able to cope with presence and clearance of material. The macrophage is considered to play a crucial role in response to implant wear ( Goodman & Gallo, 2019; Nich & Goodman, 2014; Nich *et al.*, 2016) or inhaled particles ( Ma, 2020; Riediker *et al.*, 2019) and it is accepted that the accumulation of particles does not result immediately in an adverse effect. After a phase of acute inflammation resolution and tissue repair is observed, which usually involves type 2 cytokines such as IL-10 and TGF-Beta (see Figure 1), M2 macrophages, Th2 lymphocytes and is orchestrated by so-called specialized pro-resolving mediators (SPMs). Resolution of inflammation is often disturbed and incomplete due to the presence of particles and fibrous fragments in the tissue which may lead to persistent type 2 inflammation. In the long term the ongoing remodeling in the presence of chronic low inflammation can lead to interstitial fibrosis, granuloma and tumor formation ( Ma, 2020)

This chronic, low inflammatory reaction is also the crucial mode of action causing periprosthetic osteolysis (PPOL) due to wear debris. The evidence was gathered in a series of *in-vitro* studies ( Lei *et al.*, 2019), *in vivo* experiments ( Bechtel *et al.*, 2016) and the analysis of tissues harvested during reoperations ( Gallo *et al.*, 2014). Many *in vitro* and *in vivo* studies have shown that particles generated from prosthetic materials cause immune cells such as M1 and Th1 cells to express pro-inflammatory cytokines, chemokines and other substances ( Table 1). Another piece of evidence is provided by studies that have detected the same mediators and effector cells in tissues retrieved from tissue obtained after re-operation of PPOL ( Gallo *et al.*, 2014). The osteoclast, as the most important effector cell at the bone–implant interface, is affected by the release of proteases, changed lining fluid and effects on receptor activator of nuclear factor kappa-B ligand (RANKL)-dependent pathways ( Goodman & Gallo, 2019). In concert these factors can cause local changes in differentiation, maturation, activation, and survival of this cell type, with a crucial detrimental effect on bone formation as an outcome.

**Table 1.** Characteristics of particle deposition in lung and wear in bone leading to lung fibrosis or periprosthetic osteolysis.

Parameter	Lung fibrosis/cancer	Periprosthetic Osteolysis	Key references (reviews)
Materials (particles)	Non-specific (e.g TiO _2_, carbon black, iron oxides), poorly soluble low toxicity particles (PSLT).	Non-specific (polymer, ceramics, metals), most data based on UHWMPE	*Driscoll & Borm (2020); IARC (2010);* *Merola & Affatato (2019); Mihalko *et al.* (2020)*
Relevant size range	0.5 – 5.0 micrometer Nanoparticles have higher intrinsic activity due to surface area	0.1- 10 micrometer Nanoparticles identified from ceramics and nitride coated metals	*Gibon *et al.* (2017); Morfeld *et al.* (2006); Nine *et al.* (2014); Riediker *et al.* (2019)*
Exposure	Inhalation (concentration x duration)	Internal release from implant (wear rate x duration)	*Elder *et al.* (2005); Merola & Affatato (2019)*
Particle shape	High aspect ration and > 15 µm higher response	Spherical, fibrous fragments and flakes identified (HMWPE)	*Asgharian *et al.* (2018); Nine *et al.* (2014); Oberdörster & Graham (2018)*
Deposition rate	Alveolar deposition minus dissolution and clearance	Wear rate combination of materials, components and design	*Oberdörster & Graham (2018)*
Particle distribution	Interstitial access, transport to lymph nodes	Local in tissue surrounding implant, lymph nodes	*Elder *et al.* (2005); Gallo *et al.* (2014);* *Nikula *et al.* (2001)*
Biological Response	Alveolar inflammation: neutrophil influx	Synovial inflammation (M1/M2 ratio)	*Goodman et al. (2020); Ma (2020);* *Niche & Goodman (2014); Riediker et al. (2019)*
Cytokine involvement	TNF-α, IL-8, TGF-β, IL-10	TNF-α, IL-1, IL-6, IL-18,MMP, MAPK	*Ma (2020); Piguet *et al.* (1990)* *Kandahari *et al.* (2016); Nine *et al.* (2014)*
Activation pathways	MAP kinases, NF-κB, Stat-1	MAP kinases, RANKL ,NF-κB, AP-1	*Ma (2020); Nich & Goodman (2014)* *Mbaviele *et al.* (2017); Revell (2008)*
Main effector cell(s)	Type II epithelial cells ,interstitial fibroblasts	Osteoclasts, fibroblasts	*Bissonnette *et al.* (2020); Kandahari *et al.* (2016); Mbaviele *et al.* (2017)*
Sequel responses	Type II cell Hyperplasia, Interstitial lung fibrosis, Lung cancer	Synovial plagues, Osteolysis	*Goodman & Gallo (2019); Khalil *et al.* (2007); Tzouvelekis *et al.* (2019)*
Threshold	200 cm ^2^/gram lung (rat) 10 mm ^3^/rat lung	670 mm ^3^(human) 130 mg/ml synovial fluid (human)	*Elke & Rieker (2018); Morrow (1988); Tran *et al.* (2000)*

*Abbreviations: TNF-α, tumor necrosis factor-alpha; IL-1, IL-6, IL-8 IL-18: Interleukins 1,6, 8 and 18 ; MMP, metallo-proteinases; MAPs, mitogen activated protein kinases; RANKL, receptor activator of nuclear factor kappa-B ligand ;NF-κB, Nuclear Factor- Kappa B; AP-1, Activator protein-1*

Considering the above summary of mechanisms involved, it is no surprise that particle-induced inflammation is the common driver in the adverse outcome pathway in both lung fibrosis and bone osteolysis ( Figure 1). In both cases it seems that an internal dose of small particles is reaching a level where macrophage clearance is impaired and this induces cytokine release from macrophages (M1) that leads to influx and stimulation of local cells. In the lung tissue the chronic low inflammatory response leads to cell damage, cell proliferation and fibrosis or neoplastic changes in the tissue. In the bone however, the chronic inflammatory response leads to a stimulation of OPC and a decrease of bone formation which eventually leads to PPOL. In both cases the cumulative load and initial inflammatory response caused by non-toxic particles seems to drive the chronic low inflammation, in which the key effector cells are the local M1 and M2 macrophages. Although there are major differences between the local macrophages and the influx of neutrophils and other structural effector cells (e.g. fibroblast) between bone and lung, a common AOP as depicted in Figure 1 is apparent. In both cases there is abundant evidence for a threshold dose for particles needed to reach and maintain the low chronic inflammation to cause different chronic adverse effects.

## Discussion and conclusions

This review has documented a number of parallels between two different fields of particle induced disease, that have rarely been connected before. Strong parallels are noted between the mode of action, mediators involved and the fact that the final effects are independent of material. In both endpoints (lung fibrosis and PPOL) a clear threshold is described beyond which the inflammatory response initiates. In lung particle overload the observed threshold in rats has been used to set a no observed exposure level (NOAEL) for inflammation, which in turn is important in setting occupational exposure limits for to humans exposed to so-called nuisance dusts ( ECETOC, 2013; MAK, 2014). On the other hand, based on their ability to induce (chronic) inflammation and lung cancer in the rat, the EU classification considers that such a hazard cannot be excluded in humans ( ECHA, 2017). The recent classification of TiO _2_ as a class 2 carcinogen has illustrated the limitations of EU legislation in this respect ( Riebeling *et al.*, 2020). Numerous papers have indicated that the inflammatory environment in lung particle overload in the rat may be unique in the extent of its inflammatory response and may be unique regarding the sequel of carcinogenicity ( Bermudez *et al.*, 2002; Elder *et al.*, 2005; Heinrich *et al.*, 1995; Nikula *et al.*, 2001). In addition to quantitative differences, there are qualitative differences in the inflammatory response of rats and other species – the rat inflammatory response has a marked neutrophilic component, which is largely absent in humans and non-human primates and diminished in hamsters and mice.

The case of wear particle induced PPOL is an interesting benchmark for the above considerations in the lung. The wear rate of HMWPE, as the most used liner material, is between 0.058 and 0.137 mm/year leading to theoretical lifetime between 10 and 20 years. Many studies show that the release of wear particles drives the osteolysis in bone, and a volumetric threshold of 670 mm ^3^ is a now accepted as the biological effective dose based on a systematic review of clinical data ( Elke & Rieker, 2018). In addition, clinical follow-up of patient cohorts with PPOL do not show an association with local cancer (e.g. Pijls *et al.*, 2016), whereas internal particle exposure is proven to exceed internal macrophage clearance. Although initially a slightly increased cancer mortality was found in cohorts with metal-on metal (MoM) implants ( Visuri *et al.*, 2010), later and larger studies ( Makela *et al.*, 2014; Pijls *et al.*, 2016) did not observe a total or specific cancer mortality. Perhaps the strongest evidence for absence of cancer risk is presented by a large cohort multicenter survey showing that contemporary cementless THA in young hematological disease patients after bone marrow transplantation is not associated with a higher risk for surgical complications, revision, reoperation, readmission, and mortality after 11-years follow-up ( Kim *et al.*, 2021). A second lesson may be drawn from the clinical case of THA induced osteolysis, and that is one the dose causing such effects in humans. The volumetric threshold is mainly based on outcomes in patients with HMWPE liners ( Elke & Rieker, 2018). At a density of 0.97 g/cm ^3^ this means a weight of 0.65 gram (650 mg) of PE particles and assuming that mean particle size of debris is 1 or 3 µm the total number of PE particles is 1.3 × 10 ^9 ^and 4.7 × 10 ^8^, respectively. The distribution volume of synovial fluid is estimated at an average of 5 ml based on MRI estimates in 52 subjects ( Moss *et al.*, 1998). In worst-case (no clearance, no dissolution) this would lead to a concentration of 130 mg/ml or 2.6 × 10 ^8^ particles/ml, which is many time higher than the amount observed to cause lung overload in the rat (10 mg/ml). This finding challenges the assumption that rat lung overload caused lung cancer is extrapolated as a hazard to humans ( ECHA, 2017) and adds the lack of consensus on this issue ( Driscoll & Borm, 2020).

In summary, both in lung fibrosis/cancer and PPOL, particle overload has been recognized for decades although the impact of this finding has been very different in both fields. After 30 years of its original description the debate on lung particle overload continues. The underlying mechanism(s) and differences between species and the implications for human hazard and risk assessment have only recently led to partial consensus ( Driscoll & Borm, 2020). The recent classification of TiO _2_ respirable particles by the European Chemical Agency (ECHA) as a suspected carcinogen may also impact current thinking on submicron sized TiO _2_ particles as wear from hip implants. However, as we have shown in this review PPOL after hip arthroplasty is not associated with increased risk of cancer, despite a human dose that is considered to cause particle overload. It is anticipated that the interaction between both fields can lead to further cross-fertilization of paradigms and interpretation of animal studies.

## Data availability

### Underlying data

No data are associated with article.
