# Supplementary material for: The parallels between particle induced lung overload and particle induced periprosthetic osteolysis (PPOL)
Source: Open Res Eur. 2022 Mar 9;1:16. Originally published 2021 Mar 24. [Version 2] doi: 10.12688/openreseurope.13264.2 (PMC10445866; doi:10.12688/openreseurope.13264.2)
Supplement: Peer review history

# Peer review: Reviewer response for version 2

**DOI:** 10.21956/openreseurope.15580.r28767

**Published:** 2022-05-12

**Authors:** Dominique Lison, Laeticia Perez

**Affiliations:** 1 Louvain centre for Toxicology and Applied Pharmacology (LTAP), Université catholique de Louvain, Brussels, Belgium

The author has taken our comments into account and we approve this new version of the article. We have just one minor comment for the first sentence of the part "Particle induced pathology in lung...": "Both **lung and** bone and lung" should be changed to "Both bone and lung".

Is the review written in accessible language?

Yes

Are all factual statements correct and adequately supported by citations?

Partly

Are the conclusions drawn appropriate in the context of the current research literature?

Partly

Is the topic of the review discussed comprehensively in the context of the current literature?

Yes

Reviewer Expertise:

NA

We confirm that we have read this submission and believe that we have an appropriate level of expertise to confirm that it is of an acceptable scientific standard.

# Peer review: Reviewer response for version 2

**DOI:** 10.21956/openreseurope.15580.r28768

**Published:** 2022-03-10

**Authors:** daniel Delfosse

**Affiliations:** 1 Swiss Medtech, Bern, Switzerland

Approved without any further comments.

Is the review written in accessible language?

Yes

Are all factual statements correct and adequately supported by citations?

Yes

Are the conclusions drawn appropriate in the context of the current research literature?

Yes

Is the topic of the review discussed comprehensively in the context of the current literature?

Yes

Reviewer Expertise:

20 years of experience re.the effect of wear particles from artificial joint replacment materials on the human body leading to bone osteolysis.

I confirm that I have read this submission and believe that I have an appropriate level of expertise to confirm that it is of an acceptable scientific standard.

# Peer review: Reviewer response for version 1

**DOI:** 10.21956/openreseurope.14334.r27520

**Published:** 2021-09-22

**Authors:** daniel Delfosse

**Affiliations:** 1 Swiss Medtech, Bern, Switzerland

The article describes the parallels between exposure to small non-toxic particles in human lungs and after hip replacement. It is original in the sense that no-one has made that link before and published the findings of its similarities and differences. The most important conclusions are the following:

1. For both cases, there are similar pathways leading to particle-induced inflammation, ending up either in lung fibrosis or bone osteolysis.
2. For both cases, there is a clear and distinct threshold below which the human body ist able to deal with the particle load.

This article sheds an interesting light on the two previously unlinked topics and may lead to a better understanding of the effect of particle exposure in different areas of the human body.

Is the review written in accessible language?

Yes

Are all factual statements correct and adequately supported by citations?

Yes

Are the conclusions drawn appropriate in the context of the current research literature?

Yes

Is the topic of the review discussed comprehensively in the context of the current literature?

Yes

Reviewer Expertise:

20 years of experience re.the effect of wear particles from artificial joint replacment materials on the human body leading to bone osteolysis.

I confirm that I have read this submission and believe that I have an appropriate level of expertise to confirm that it is of an acceptable scientific standard.

# Peer review: Reviewer response for version 1

**DOI:** 10.21956/openreseurope.14334.r26862

**Published:** 2021-05-26

**Authors:** Dominique Lison, Laeticia Perez

**Affiliations:** 1 Louvain centre for Toxicology and Applied Pharmacology (LTAP), Université catholique de Louvain, Brussels, Belgium

This review highlights similarities of the pathological reactions induced by foreign particulate materials which can appear in two apparently unconnected biomedical fields, i.e. lung toxicology and orthopedics. The idea is that the tissue reaction induced by inhaled poorly soluble low-toxicity particles (PSLT) in the lung shares pathophysiological pathways with the osteolytic reaction (PPOL) induced by wear debris materials around bone implants such as hip prosthesis. The exercise is original and bears a potential for cross-fertilization between both fields. Specific avenues for possible cross-fertilization are not covered.

 The author might wish to consider the following comments and suggestions.

**Major comments.**

1. While a notion of threshold dose for particulate materials has been identified for both PPOL and PSLT lung reactions, the role of overload is less apparent for PPOL than in the lung. Thus, threshold (in bone) is not necessarily synonymous to particulate overload.
2. The tissue reaction induced by wear particulates does not occur in the bone but in the joint (synovial fluid).
3. The comparison between bone implants and lung transplantation (end of the plain language summary) is probably not relevant or useful.
4. In Morrow’s original theory, overload is initiated from a deposited dose corresponding to 6% of macrophage volume, not 1/6 ^th^ of its volume. This volume corresponds to 1 µL/gram lung (not 0.1 µL) (see Oberdörster *et al.* 1992 ^1^). Please make clear that the estimated surface area threshold of 200 cm² is per lung (and not per gram lung).
5. Figure 1 : particle accumulation (not cumulation) is a consequence of clearance impairment, and should not appear as the initial event.
6. Regarding IL-10 and TGF-b, it might be worth mentioning that those anti-inflammatory (immunosuppressive) cytokines, are also strong pro-fibrotic molecules. Thus, the persistence of the anti-inflammatory reaction (resulting from the persistence of the particulate material) fuels the fibrotic reaction (see Huaux 2018 ^2^).
7. It might also be useful to note that osteoclasts are derived from the monocyte/macrophage lineage by the action of macrophage colony stimulating factor (M-CSF) and receptor activator of nuclear factor-kB ligand (RANKL) ^3^.
8. Table 1 : the line on particle shape (and references) is probably not relevant here, as we are not dealing with high aspect ratio materials.
9. Discussion and conclusions – first para : the last sentence should be supported by a reference.
10. Discussion and conclusions : the threshold value (and unit) of 10 mg/ml for lung overload is unclear. The threshold dose for overload is usually expressed as an amount (in mass, volume or surface area), not as a concentration. Not sure PPOL and PSLT can be compared on this basis.

**Minor comments**.

 Abstract – background : read “titanium dioxide”.

 Abstract – conclusions : please specify relevance for what.

 Introduction – first para : read “pathogenesis” instead of “etiology”.

 Introduction – second para : read “diesel exhaust particles”.

 Introduction – end second para : read “it is now suggested that particle surface dose (omit "rate") might be a better …”

 Introduction - last para – second line : delete “and humans”.

 Page 4, last para, first line : delete “and lung”.

 Page 5, second para left : read “ has illustrated the limitations of a hazard-based CLP legislation”. It would also be useful to briefly indicate which limitations.

 Reference Mbaviele (2017) should be Mba laviele (2017).

 Acronyms should be defined in full at first appearance in the text.

 The text might also need some polishing to remove or add words where needed.

Is the review written in accessible language?

Yes

Are all factual statements correct and adequately supported by citations?

Partly

Are the conclusions drawn appropriate in the context of the current research literature?

Partly

Is the topic of the review discussed comprehensively in the context of the current literature?

Yes

Reviewer Expertise:

Inhaled particles toxicology.

We confirm that we have read this submission and believe that we have an appropriate level of expertise to confirm that it is of an acceptable scientific standard, however we have significant reservations, as outlined above.

## References

[1] : Volumetric loading of alveolar macrophages (AM): a possible basis for diminished AM-mediated particle clearance. *Exp Lung Res* .18(1) : 10.3109/01902149209020653 87-104 10.3109/01902149209020653 1572327

[2] : Emerging Role of Immunosuppression in Diseases Induced by Micro- and Nano-Particles: Time to Revisit the Exclusive Inflammatory Scenario. *Front Immunol* .2018;9: 10.3389/fimmu.2018.02364 2364 10.3389/fimmu.2018.02364 30510551PMC6252316

[3] : The Alternative Faces of Macrophage Generate Osteoclasts. *Biomed Res Int* .2016;2016: 10.1155/2016/9089610 9089610 10.1155/2016/9089610 26977415PMC4761668
